# Diet, Lifestyles, Family History, and Prostate Cancer Incidence in an East Algerian Patient Group

**DOI:** 10.1155/2016/5730569

**Published:** 2016-10-19

**Authors:** Somia Lassed, Cláudia M. Deus, Nuno Lourenço, Abderrezak Dahdouh, Albert A. Rizvanov, Paulo J. Oliveira, Djamila Zama

**Affiliations:** ^1^Unité de recherche Valorisation des Ressources Naturelles, Molécules Bioactives et Analyses Physicochimiques et Biologiques (VARENBIOMOL), Université des Frères Mentouri, Constantine, 25000 Constantine, Algeria; ^2^Laboratoire de Physiologie Animale, Département de Biologie Animale, Faculté des Sciences de la Nature et de la Vie, Université des Frères Mentouri, Constantine, 25000 Constantine, Algeria; ^3^Center for Neuroscience and Cellular Biology (CNC), University of Coimbra, UC Biotech Building, Biocant Park, 3060-197 Cantanhede, Portugal; ^4^Department of Informatics Engineering, Center for Informatics and Systems of University of Coimbra (CISUC), Polo II-Pinhal de Marrocos, 3030 Coimbra, Portugal; ^5^Clinic of Urology-Nephrology and Kidney Transplant Daksi, 25000 Constantine, Algeria; ^6^Institute of Fundamental Medicine and Biology, Kazan Federal University, Kazan, Russia

## Abstract

Prostate cancer (PC) is the fourth most common cancer in men and the sixth leading cause of death in Algeria. To examine the relationship between lifestyle factors, including diet, and family history and PC risk, a case-control study was performed in an eastern Algerian population, comprising 90 patients with histologically confirmed PC and 190 controls. Data collection was carried out through a structured questionnaire and statistical analysis was performed to evaluate the different variables. The data showed that consumption of lamb and beef meat and high intake of animal fat and dairy products increased PC risk. Seven to thirteen vegetables servings per week and fourteen or more servings decreased PC risk by 62% and 96%, respectively. Seven to fourteen fruit servings per week decrease PC risk by 98%. Green tea consumption reduced the risk of PC but the results were statistically borderline. Increased risk was observed for individuals with family history of PC in first and in second degree. A positive strong association was also found for alcohol and smoking intake and a dose-response relationship existed for quantity and history of smoking. This study suggests that dietary habits, lifestyle factors, and family history have influence on the development of PC in Algerian population.

## 1. Introduction

After lung cancer, prostate cancer (PC) is the most common cancer among men, with nearly a million new cases diagnosed worldwide [1]. PC incidence varies more than 25-fold worldwide; the rates are highest in Australia/New Zealand, Northern America, and Western and Northern Europe (ASR 111.6, 97.2, 94.9 and 85.0 per 100,000, resp.) and relatively high in certain regions such as the Caribbean (79.8), Southern Africa (61.8), and South America (60.1), but it is low in Asian population with estimated rates of 10.5 and 4.5 in Eastern and South-Central Asia [2]. According to GLOBOCAN 2012 database, PC in Algeria was the fourth most common cancer in men with 8.8 cases per 100,000 and the sixth leading cause of death with 4.9 deaths per 100,000. The incidence of PC in Algeria is considered as low when compared with some western countries such as Norway and Sweden (129.7 and 119.0 cases diagnosed and approximately 18 deaths per 100,000, resp.), but it is still high compared to many Asian countries especially China, Korea, and Bhutan (5.3, 3.2, and 1.2 cases diagnosed and 2.5, 1.3, and 0.7 deaths per 100,000, resp.) [2]. Numerous epidemiological studies showed that the incidence of PC in Asian countries is low compared to the West. However, this incidence rapidly increases in Asian immigrants that have assimilated western diet and way of living, replacing soy, tea, fish, fruits, and vegetables consumption with red meat and fat-rich food [3]. Also, a study conducted in Western Australia described that the risk of PC and in particular aggressive PC increases significantly in about 80% of men who consume a western style diet [4]. The difference in PC rates may result in possible dietary, lifestyle, and environmental links to the development and progression of PC [5, 6]. Many epidemiological and case-control studies disclosed that some dietary factors such as animal fat [7], dairy products [8], and red meat [9] may increase the risk of PC [7–9], whereas intake of vegetables and fruit [10], fish [11], and green tea [12] appears to be potentially protective [10–12]. The aim of this study was to examine the relationship between dietary habits, family history of PC, alcohol, and smoking and PC risk in an East Algerian population.

## 2. Materials and Methods

### 2.1. Data Collection

The present study was approved by the Ethics Committee of the EHS Daksi, which certified that the data collection was performed at the Department of Urology and Renal Transplant without any risk for patients. Data were derived from a case-control study of PC, performed between 2011 and 2013 in the east of Algeria. From 100 cases of histologically confirmed PC, followed at the service of urology and at the emergency department in Clinic of Urology-Nephrology and Kidney Transplant Daksi, Constantine, one patient refused to participate and 9 were too ill to be interviewed. The cohort investigated consisted of 90 patients with a median age of 68.87 ± 0.73, ranging from 50 to 88 years. The total Gleason score, serum PSA level at diagnosis, and primary treatment taken for each case were obtained from the medical record. Controls were men, residing in the same geographical area of cases without either PC or prostate diseases. Those with other malignancies were also excluded. Among 200 men selected, 10 refused to participate for different reasons. The final control group consisted of 190 men with a median age of 67.13 ± 0.72, ranging from 50 to 88 years. Sixty-one men were treated at the hospital for some urologic diseases (35 had kidney stone, 25 had urinary infection, and 10 had other diseases), 14 were men from the hospital staff invited to participate, and 115 were men selected in the blood sample collection room of the Laboratory of Biochemistry of Establishment Public Hospital of Chelghoum Laid city.

Data collection was carried out through an interview, using structured questionnaire, including information on sociodemographic characteristics, dietary habits, family history of PC, smoking, and alcohol consumption (see Supplementary Material available online at http://dx.doi.org/10.1155/2016/5730569). All the interviews were filled face-to-face with case and control individuals. Questionnaire on dietary habits included questions about the frequency of intake of different food items during the past years. The first item was about the number of meals per day, followed by questions about the consumption of fish and red meat (lamb and beef), by using the question “how many times per week or month,” and consumption of animal fat (from red meat) or dairy products (milk, cheese, and yogurt) by using the expressions “never, small, medium or great part.” In the latter, “small or medium part” of dairy products meant one cup of milk or/and some other product per day, and “great part” meant more than 2 cups of milk per day or/and a large amount of other product. Another question was related to total fruit and vegetables consumption by using the question “how many times per week. The last food-related item is regarded as green tea consumption by using the question “how many cups per day.” Participants were also asked about alcohol and smoking employing the terms “non-users,” “former users,” and “current users.” Former users were defined as those who had consumed alcoholic beverages or tobacco earlier in their life but not during the last years and current users were considered as those who actually still consume alcoholic beverages or tobacco. The total tobacco consumption was determined using pack-years unity. The original questionnaire is presented as supplementary material. All the participants were asked about their dietary habits 10 years prior to the study because the clinical PC has a long latency phase, during which the disease is histologically present and the remote dietary intake may be more important than recent dietary intake when predicting the risk of PC [13].

### 2.2. Statistical Analyses

The data from 90 PC cases and 190 controls were analyzed using Graph Pad Prism version 7. Firstly we started by analyzing if the samples follow a normal distribution. We relied on the Shapiro-Wilk test at a significance level of 0.05. The results showed that the data did not follow a normal distribution so all the tests from here on will assume nothing about the distribution of the data; that is, we will rely on nonparametric tests to analyze the data. To check whether there were statistically significant differences between the control and cases groups, we used the Mann–Whitney test for continuous variables, such as PSA values.

The remainder of the variables (different types of food consumption, family history of PC, smoking habits, and alcoholic consumption) were grouped into categories, corresponding to the different possible answers. The individual frequency of the answers was measured and stored in a contingencies table. All tables were analyzed using the chi-square test. To compute the confidence intervals of the odds ratio (OR) we used on the Woolf logit method. *p*_trend_ values were determined using the chi-square test for trend.

## 3. Results

The Gleason score at diagnosis was between 5 and 7 in 38% of cases and it was between 8 and 10 in 62% of cases. 12% of cases were oriented toward prostatectomy as initial treatment and 88% were oriented to start hormone therapy. There were no statistical significant differences in age between cases and controls (*p* = 0.101). Moreover, the nonparametric test Kolmogorov-Smirnov showed that the ages of the two groups (control and cases) were taken from the same distribution. The median prostate-specific antigen (PSA) level was higher in diseased individuals than in controls (*p* < 0.0001). All the participants were living in the same geographical area distributed by different cities in the east of Algeria (Table 1).

Table 2 shows the odds ratio and the relative risk for the relationship between the intake of different food items and PC. When comparing men who ate more than three meals per day to men who consumed only two to three meals per day, no statistically significant associations were observed between numbers of meals consumed per day and PC risk. Also, no statistical association was found between fish consumption and PC, when comparing men who ate fish more than three times per month to those who had fish in their meals less than 2 times per month. A significant relationship between red meat consumption and PC risk was observed. A strong positive association (*p* < 0.0001) between lamb consumption and PC was found. The odds ratio and the relative risk increased significantly with the increased lamb meat meals consumed per month. Compared to those who never or rarely consumed lamb, they were, respectively, 3.33 (1.74 to 6.37) and 2.46 (1.49 to 4.06), *p* = 0.0002, in those who consumed lamb between one to two times per month and were 4.99 (2.36 to 10.53) and 3.10 (1.84 to 5.24), *p* < 0.0001, in those who consumed lamb three times or more per month. A positive association was also found between beef consumption and PC (*p*_trend_ = 0.013), but it was only significant in men who ate beef three times or more per month. Concerning animal fat derived from red meat consumption, a strong positive correlation was found (*p*_trend_ < 0.0001). Increased risk was observed for men with high intake of animal fat compared to those who never eat animal fat or just normally consumed a small part relative to the total of food intake daily [OR 7.38 (95% CI 3.87 to 14.06) and RR 3.02 (95% CI 2.17 to 4.20); *p* < 0.0001]. The dairy product intake was also associated with PC risk. The risk increased significantly in men with high intake of dairy products compared to those who consumed just a small or medium part daily. Inverse strong association was observed between total fruit and total vegetables consumption and PC risk (*p*_trend_ < 0.0001). The odds ratio and the relative risk decreased significantly with increased fruit and vegetables consumed per week. The results obtained showed that PC is less common in men who consumed vegetables 7 to 13 times per week and much less common for those who intake vegetables 14 or more times per week and fruit 7 to 14 times per week. Results concerning green tea consumption showed that PC risk decreased with the number of cups of green tea consumed per day. However, these results were not significant (*p*_trend_ = 0.07), and OR and RR were, respectively, 0.64 (95% CI 0.36 to 1.15) and 0.74 (95% CI 0.48 to 1.12), *p* = 0.1569, for those who drank 1 to 3 cups per day, and they were 0.40 (95% CI 0.08 to 1.92) and 0.51 (95% CI 0.14 to 1.82), *p* = 0.2025, for individuals who had consumption of more than 3 cups per day.

The results in Table 3 indicate that a higher proportion of cases had a family history of PC (22%) compared to controls (4%). A statistically significant association between family history of PC in first degree (father or brother) and PC risk was observed. Men with a family history of PC in second degree (grandfather or uncle) were also at higher risk of getting PC. A positive strong correlation was observed between PC incidence and alcohol consumption (*p* < 0.0001). The odds ratio and the relative risk for former users compared to nonusers were, respectively, 5.53 (95% CI 2.68 to 11.42) and 2.51 (95% CI 1.84 to 3.40). Also, smoking status showed a potent positive association between smoking and the risk of getting PC (*p*_trend_ < 0.0001). The odds ratio and relative risk for former smokers compared to nonsmokers were, respectively, 3.17 (95% CI 1.76 to 5.69) and 2.27 (95% CI 1.47 to 3.50) and, for current smokers, the values were, respectively, 4.05 (95% CI 1.84 to 8.89) and 2.60 (95% CI 1.57 to 4.31). According to total tobacco consumption, we found a dose-response relation to PC risk which was 3.56-fold higher in men who consumed more than forty pack-years (more than 292 kg of tobacco) in their life (Table 3).

## 4. Discussion

In this study, controls and PC-positive patients were collected in the same period and interviewed face-to-face using a structured questionnaire to decrease the probability of bias. Cases were interviewed only after the confirmation of their disease and controls were selected carefully and matched to disease cases based on age and region of residence. The use of past 10 years' dietary history is justified by the fact that PC has a long latency period (10 to 15 years) [13] and because dietary changes may have occurred after diagnosis of the disease, thus not reflecting the typical diet consumed before the onset of PC. Still, since this is a case-control study we cannot exclude a certain degree of recall bias [14].

The number of meals taken per day, which can be an indicator of total energy intake, showed no association with the risk of PC in this study. Theoretically, high energy intake can play a relevant role in several neoplasms since it may stimulate the sympathetic nervous system and basal metabolism leading to increased IGF-1 release which increases cell proliferation automatically through the stimulation of mitosis [15]. Several case-control studies reported an association [16–18] or no association [19, 20] between increased PC risk and high energy intake.

Numerous epidemiological studies examined the relationship between fish consumption and PC. Although many suggested risk reduction associated with high intake, others are contradictory. A follow-up of 47,882 men during 12 years in the Health Professionals Study found that the consumption of more than three times per week of fish was associated with a reduced risk of PC. The strongest association was found for metastatic cancer (RR 0.56; 95% CI 0.37 to 0.86, compared with infrequent consumption, i.e., less than twice per month) [11]. Similarly, five or more servings of fish per week versus less than once was associated with a significant reduction in PC risk [21]. Also, a recent study suggested that prostate tissue *ω*-3 fatty acids, especially eicosapentaenoic acid (EPA), are protective against PC progression in men with low risk for that disease [22]. However a meta-analysis study provided no strong evidence of a protective association of fish consumption with PC incidence [23]. No association was observed in our study between fish consumption and PC, which may be explained by the low levels of fish consumption, compared to other studies cited [11, 21].

Our study showed a strong association between red meat and high animal fat consumption and PC. Several previous studies examined the relationship between red meat and PC risk with conflicting results. Some studies suggested a positive association [9, 24, 25], whereas others have found no association [26, 27]. Among studies which suggested a positive association, a case-control study found that eating red meat (beef, lamb, or pork) at least 5 times per week increased the risk of PC incidence by 2.5-fold [9], which was confirmed by further studies [24]. The increased risk may stem from triggering of carcinogenesis by high fat content, formation of carcinogenic heterocyclic amines (HCAs) during cooking at high temperature, carcinogenic N-nitroso compounds (NOCs), and the promotion of carcinogenesis by heme iron [25, 26], although infection by tumorigenic pathogens has been increasingly denoted as possible cause [28]. Animal fat was positively associated with PC risk in this study, in line with previous works [7, 29, 30] but not others [31, 32]. The prospective cohort study of Giovannucci et al. concluded that red meat and total fat consumption was directly related to the risk of advanced PC and the association of the latter was primarily due to animal fat. However, fat from dairy products (with the exception of butter) or fish was unrelated to the risk of PC [7]. Also in a prospective study, men in the lower tercile of saturated fat consumption compared to those in the upper tercile had three times the risk of dying from PC [29]. The link between dietary fat content and the development of PC is possibly related to the increase in sex hormone and IGF-1 concentration [33], alteration of cell membrane function, and modulation of metabolic processes [34] or to increased oxygen radicals generation [35].

Total dairy product intake was also positively associated with the risk of PC in this study. The relationship between PC and dairy products showed significant positive association [8, 36, 37], overall null association [38, 39], or an inverse association [40]. In the epidemiologic follow-up study, results were similar to ours, showing that men in the highest tercile for dairy food intake had a relative risk of 2.2 (95% CI 1.2 to 3.9; *p*_trend_ = 0.05) compared with men in the lowest tercile [8]. However, in the majority of other studies the relative risk was lower, 1.63 (95% CI 1.14 to 2.32) and *p*_trend_ = 0.01, in a population based prospective study performed with 43,435 Japanese men [36] and 1.26 (95% CI 1.04 to 1.51) and *p*_trend_ = 0.03 in a large prospective study from Finland [37]. Several potential mechanisms explain the relationship between dairy product intake and the increased PC risk. First, the high level of calcium or phosphate content in plasma may lower intracellular bioactive metabolite of vitamin D, 1,25 dihydroxycholecalciferol concentration, [41] which is described to have an antiproliferative effect [42]. Second, higher intake of milk and calcium has been associated with increased plasma levels of IGF-1 [43]. Third, estrogen content in milk has been suggested also as possible explanation [44].

Among all food items studied, our study showed that total vegetables and fruit consumption presented a statistically significant protective effect on PC. These results support that high consumption of vegetables and fruit are associated with decreased risk of PC and are in agreement with the findings of a case-control study performed in Montevideo [10]. Nevertheless the finding in this context remains contradictory. Some studies suggested that total fruit or vegetable consumption may not exert a protective role [24, 45]. However, in another study a large fruit intake was associated with increased risk of PC [46].

Green tea polyphenols, especially catechins, inhibit carcinogenesis by different mechanisms [47] and in the majority of epidemiological studies a significant decrease in the risk of PC incidence was observed with increasing intake of green tea. In a case-control study in Southeast China, a consumption of 3 cups of green tea daily lowered the risk of PC [12]. In a large cohort study in Japan, only 5 or more cups per day reduced the risk of advanced PC [48]. Agreeing with some studies [49, 50], our results did not confirm the protective effect of green tea consumption. This can be explained by the fact that green tea is not a popular beverage in Algeria. The studied population reflected the fact that most of the studied individuals rarely or never drink green tea.

The family history of PC was another factor risk in the Algerian population studied. 22% of PC cases had a family history of PC, against only 4% of controls. Family history of PC has been examined in many studies and in different populations, with most studies finding a positive association [51–53]. Steinberg et al. observed increased PC risk among men whose first or second degree relatives also had PC [52]. In a multicenter case-control study performed in the US and Canada in Afro-American, Whites, and Asian-Americans, 13% of cases and 5% of controls reported a father, brother, or son with PC and family history association with a statistically significant 2- to 3-fold increase in risk in each of the three ethnic groups [53]. Also, in a recent study in Brazil, family history was also a PC risk factor [51]. In addition to the positive association found between family history and PC risk, the latter increased with the number of relatives affected [52].

Our results suggest that alcohol intake is associated with increased PC risk. No current users were found in the population studied since cases and control individuals never drink alcohol or just drank a few times during their youth, with the rest (29% cases and 7% controls) being former users. Most studies showed no association between alcohol intake and PC [54–56] with a few studies reporting an inverse association [57, 58]. In a follow-up study of alcohol and PC, a significant inverse association was shown between distant past heavy drinking (25 drinks/week) and PC [58]. Similarly, an inverse association was observed in another study with the total number of drinking years but no association was found with lifetime intake of total and beverage specific ethanol [57]. In these studies, the protective role of alcohol observed has been interpreted by the finding that alcohol may increase metabolic clearance of testosterone [59]. Nevertheless, our study is comparable with many others supporting the hypothesis that alcohol increases PC risk. In a recent case-control study, current alcohol intake was not associated with PC but lifetime intake increased significantly the risk of both aggressive and nonaggressive PC [60] while, in the cohort of US health professionals, men who drank large amounts of alcohol in a short time were also found to be at higher risk of PC [61]. Other studies suggested that heavy alcohol consumers or alcohol abusers have higher PC incidence, with 4 alcoholic drinks per day being associated with about a 21% increased risk of PC [62]. In our study, we did not have enough information from all cases and controls to examine the association between the amount and the period of alcohol intake and PC risk.

In contrast to several studies which did not support that smoking is a factor risk of PC [17, 24, 55], we found out that smoking was associated with increased risk. Our results support the finding of other studies which found a positive association between smoking and PC risk. In the study of Plaskon et al. current smokers were associated with an increased risk in contrast to former smokers, with a dose-response relation noted between number of pack-years smoked and PC risk [63]. In another study, the relative risk for PC was significantly elevated among cigarette smokers but no clear dose-response relation for was observed [64]. Another study found that early (before age 30), late (within recent 10 years), and lifetime cumulative smoking history were unrelated to the risk of total PC. While men who had smoked 15 or more pack-years of cigarettes within the preceding 10 years were at higher risk of distant metastatic and fatal PC, the excess risk among smokers was eliminated after 10 years of quitting [65]. Two pathogenic mechanisms were proposed to link cigarette smoking and increased PC risk. The first one is related to carcinogenic substances found in cigarettes such as cadmium which can indirectly induce PC through the interaction with androgen receptor [66]. A second factor relates to the effect of smoking on sex hormones level. Male smokers are reported to have elevated level of circulating testosterone [67].

## 5. Conclusion

Our study indicates that dietary habits, family history of PC, alcohol intake, and smoking may influence the development of PC in East Algerian population. Risk factors for PC appeared to be the intake of red meat, high consumption of animal fat and dairy product, family history of PC, alcohol intake, and smoking. A protective effect was found for high intake of fruit and vegetables. Green tea reduced, although not statistically significant, the risk of PC. However the number of meals intake per day and fish consumption was found to be independent factors for PC.

## Supplementary Material

The supplementary material includes the French (original) and English version of the questionnaire handed to the individuals which participated in this study.

## Figures and Tables

**Table 1:** Prostate cancer cases and controls characterization.

	Cases, *n* (%)	Controls, *n* (%)	*p* value^b^
No. of subjects	90	190	
Mean age ± SD	68.87 ± 0.73^a^	67.13 ± 0.72^a^	0.1012
Mean PSA ± SD (ng/mL)	122 ± 22.04	1.71 ± 0.10	<0.0001^*∗∗∗∗*^
Gleason score			
5–7	34 (38)	—	—
8–10	56 (62)	—	—
Primary treatment			
Prostatectomy	11 (12)	—	—
Hormone therapy	79 (88)	—	—
City of living			
Constantine	34 (38)	60 (32)	—
Mila	16 (18)	43 (23)	—
Guelma	4 (4)	10 (5)	—
Setif	5 (6)	12 (6)	—
Oum El Bouaghi	10 (11)	15 (8)	—
Jijel	8 (9)	19 (10)	—
Skikda	6 (7)	14 (7)	—
Bejaia	2 (2)	5 (3)	—
Tebassa	3 (3)	8 (4)	—
M'Sila	2 (2)	4 (2)	—

^a^Values are expressed as mean ± SD.

^b^Based on Mann-Whitney; PSA: prostate-specific antigen; ^*∗∗∗∗*^ *p* < 0.0001.

**Table 2:** Association between the amount and kind of different food items and the risk of prostate cancer.

	Cases, *n* (%)	Controls, *n* (%)	Odds ratio^a^	95% CI^a^	Relative risk^a^	95% CI^a^	*p* value^a^
*Number of meals/day*							
2 to 3 meals	70 (78)	158 (83)	1				
>3 meals	20 (22)	32 (17)	1.41	0.75–2.63	1.25	0.84–1.86	0.3238
*Fish, time/month*							
0–2	55 (61)	118 (62)	1				
≥3	35 (39)	72 (38)	1.04	0.62–1.74	1.03	0.72–1.45	0.8957
*Red meat, time/month*							
*Lamb*							
0	17 (19)	95 (50)	1				
1-2	40 (44)	67 (35)	3.33	1.74–6.37	2.46	1.49–4.06	0.0002^*∗∗∗*^
≥3	25 (28)	28 (15)	4.99	2.36–10.53	3.10	1.84–5.24	<0.0001^*∗∗∗∗*^
*p*_trend_^b^			<0.0001^*∗∗∗∗*^				
*Beef*							
0	16 (18)	57 (30)	1				
1-2	39 (43)	81 (43)	1.71	0.87–3.36	1.48	0.89–2.45	0.1394
≥3	35 (39)	52 (27)	2.39	1.19–4.83	1.83	1.11–3.03	0.0170^*∗*^
*p*_trend_^b^			0.013^*∗*^				
*Animal fat*							
Never or small part^1^	37 (41)	127 (67)	1				
Medium part^1^	10 (11)	43 (23)	0.79	0.36–1.74	0.83	0.44–1.56	0.7020
Great part^1^	43 (48)	20 (10)	7.38	3.87–14.06	3.02	2.17–4.20	<0.0001^*∗∗∗∗*^
*p*_trend_^b^			<0.0001^*∗∗∗∗*^				
*Dairy product*							
Small or medium part^2^	45 (50)	144 (76)	1				
Great part^3^	45 (50)	46 (24)	3.13	1.84–5.31	2.07	1.49–2.88	<0.0001^*∗∗∗∗*^
*Total fruit, time/week*							
<2	17 (19)	4 (2)	1				
2–6	59 (66)	34 (18)	0.40	0.12–1.31	0.78	0.60–1.01	0.198
7–14	14 (15)	152 (80)	0.02	0.00–0.07	0.10	0.06–0.17	<0.0001^*∗∗∗∗*^
*p*_trend_^b^			<0.0001^*∗∗∗∗*^				
*Total vegetables, time/week*							
<7	18 (20)	7 (4)	1				
7–13	60 (67)	61 (32)	0.38	0.14–0.98	0.68	0.50–0.93	0.048^*∗*^
≥14	12 (13)	122 (64)	0.03	0.01–0.10	0.12	0.06–0.22	<0.0001^*∗∗∗∗*^
*p*_trend_^b^			<0.0001^*∗∗∗∗*^				
*Green tea consumption, cups/day*							
<1	67 (75)	122 (64)	1				
1–3	21 (23)	59 (31)	0.64	0.36–1.15	0.74	0.48–1.12	0.1569
>3	2 (2)	9 (5)	0.40	0.08–1.92	0.51	0.14–1.82	0.2025
*p*_trend_^b^			0.07				

95% CI = 95% confidence interval. ^a^Determined using the chi-square test. ^b^Determined using the chi-square test for trend; ^*∗*^ *p* < 0.05, ^*∗∗∗*^ *p* < 0.005, and ^*∗∗∗∗*^ *p* < 0.0001.

^1^Relative to the total of daily food intake. ^2^One cup of milk or/with some other product (cheese, yogurt) per day. ^3^More than 2 cups of milk per day or/with a big quantity of other product (cheese, yogurt).

**Table 3:** Association between family history of prostate cancer, alcohol consumption, and smoking habits and the risk of prostate cancer.

	Cases, *n* (%)	Controls, *n* (%)	Odds ratio^a^	95% CI^a^	Relative risk^a^	95% CI^a^	*p* value^a^
*Family history of prostate cancer*							
Absent	70 (78)	182 (96)	1				
In first degree	11 (12)	3 (1)	9.53	2.58–35.20	2.82	2.01–3.96	0.0002^*∗∗∗*^
In second degree	9 (10)	5 (3)	4.68	1.51–14.45	2.31	1.49–3.58	0.0063^*∗∗*^
*p*_trend_^b^			<0.0001^*∗∗∗∗*^				
*Alcohol status*							
Nonusers	64 (71)	177 (93)	1				
Former users	26 (29)	13 (7)	5.53	2.68–11.42	2.51	1.84–3.40	<0.0001^*∗∗∗∗*^
*Smoking status*							
Nonsmokers	22 (24)	99 (52)	1				
Former smokers	50 (56)	71 (37)	3.17	1.76–5.69	2.27	1.47–3.50	0.0001^*∗∗∗*^
Current smokers	18 (20)	20 (11)	4.05	1.84–8.89	2.60	1.57–4.31	0.0006^*∗∗∗*^
*p*_trend_^b^			<0.0001^*∗∗∗∗*^				
*Total tobacco consumption, unity of pack-years*							
0	22 (24)	99 (52)	1				
1–9	9 (10)	20 (11)	2.02	0.81–5.04	1.70	0.88–3.30	0.1326
10–19	15 (17)	27 (14)	2.50	1.14–5.46	1.96	1.12–3.42	0.0311^*∗*^
20–29	11 (12)	23 (12)	2.15	0.91–5.05	1.78	0.96–3.29	0.0964
30–39	9 (10)	8 (4)	5.06	1.75–14.59	2.91	1.62–5.23	0.0033^*∗∗*^
≥40	24 (27)	13 (7)	8.30	3.66–18.83	3.56	2.28–5.57	<0.0001^*∗∗∗∗*^
*p*_trend_^b^			<0.0001^*∗∗∗∗*^				

95% CI = 95% confidence interval. ^a^Determined using the chi-square test. ^b^Determined using the chi-square test for trend; ^*∗*^ *p* < 0.05, ^*∗∗*^ *p* < 0.01, ^*∗∗∗*^ *p* < 0.005, and ^*∗∗∗∗*^ *p* < 0.0001.
