# Supplementary material for: Diet, Lifestyles, Family History, and Prostate Cancer Incidence in an East Algerian Patient Group
Source: Biomed Res Int. 2016 Oct 19;2016:5730569. doi: 10.1155/2016/5730569 (PMC5130556; doi:10.1155/2016/5730569)
Supplement: Supplementary file 1 — The supplementary material includes the French (original) and English version of the questionnaire handed to the individuals which participated in this study. [file 5730569.f1.pdf]

Université Mentouri 1 – Constantine
Département de biochimie

Questionnaire concernant l'alimentation, style de vie et les antécédents réalisé dans le cadre de doctorat

1. **Informations personnel**

Date et lieu de naissance: Adresse:

Poids: Taille:

Travail ou ancien travail:

Niveau d'éducation:

2. **Maladies actuelles ou anciennes:**

3. **Informations médicales (concernant les malades du cancer de la prostate):**

- L'âge de diagnostique:
- PSA:
- Stade de la maladie:
- Traitement médicamenteux:
- Les analyses effectuées:
- Autres informations:

4. **Antécédents familiaux au cancer de la prostate:**

☐ RAS

☐ Père

☐ Grand-père

☐ Frère nombre:

☐ Fils nombre:

☐ Oncle nombre:

☐ Cousin nombre:

5. **Alimentation**

- **Nombre de repas/jour:**

☐ 1 ☐ 2 ☐ 3 ☐ plus

- **Type de pain:**

☐ Galettes ou pain complets ☐ Pain ordinaire

- **Consommation de poisson, nombre de fois/mois :**

☐ 0 ☐ <1 ☐ 1 ☐ 2 ☐ 3 ☐ 4 ☐ > 1 fois par semaine

- **Consommation de viande rouge, nombre de fois/mois:**

  - Viandes de moutons:

☐ 0 ☐ <1 ☐ 1 to 2 ☐ 3 to 4 ☐ 4 ☐ > 1 fois par semaine

  - Viandes de veaux :

☐ 0 ☐ <1 ☐ 1 to 2 ☐ 3 to 4 ☐ 4 ☐ > 1 fois par semaine

- **Consommation des graisses saturées (d'origine animale):**

☐ Pas de consommation ☐ Une petite consommation ☐ Consommation moyenne

☐ Consommation élevée

- **Consommation des produits laitiers:**

☐ Une petite consommation (a) ☐ Consommation moyenne (b)

☐ Consommation élevée (c)

(a) Une seule tasse de lait ou d'autres produits (fromage, yaourt ..) par jour
(b) Une tasse de lait avec un autre produit (fromage, yaourt ..) par jour
(c) Plus de 2 tasses de lait par jour et / ou avec une grande quantité d'un autre produit (fromage, yaourt ..).

- **Consommation des fruits, fois/semaine:**

☐ < 2 ☐ 2 to 6 ☐ 7 to 14

- **Consommation des légumes, fois/semaine:**

☐ < 7 ☐ 7 to 13 ☐ ≥ 14

- **Consommation de thé vert, tasse/jour:**

☐ 0 ☐ < 1 ☐ 1 to 3 ☐ > 3

6. **Habitudes toxiques** :

- Alcool

☐ Jamais ☐ Utilisateur actuel (pour combien d'années et combien de boisson / jour)

☐ Ancien utilisateur (bu et arrêté pendant combien d'années et combien de boisson / jour)

- Tabac

☐ Non fumeur

☐ Fumeur actuel (Nombre de cigarette ou paquet par jour et pendant combien d'années)

☐ Ancien fumeur (Nombre de cigarette ou paquet par jour et pendant combien d'années et arrêté depuis quand)

(Translated from an original document in French)

Food frequency questionnaire (FFQ)

1. **Personal information**

Date and place of birth: **Address:**

Weight: **Height:**

Occupation or former occupation:

Level of education:

2. **Current or past diseases:**

3. **Family history of prostate cancer:**

☐ **Father**

☐ **Grandfather**

☐ **Brothers** **How many:**

☐ **Sons** **How many:**

☐ **Uncles** **How many:**

☐ **Cousins** **How many:**

4. **Dietary intake**

- **Number of meals/day:**

☐ **1** ☐ **2** ☐ **3** ☐ **more**

- **Kind of bread :**

☐ **Complete bread** ☐ **Plain bread**

- **Fish consumption, number of time/month:**

☐ **0** ☐ **<1** ☐ **1** ☐ **2 to 3** ☐ **4** ☐ **> 1 time per week**

- **Red meat consumption, number of time/month:**
    - **Lamb:**

☐ **0** ☐ **<1** ☐ **1 to 2** ☐ **3 to 4** ☐ **4** ☐ **> 1 time per week**

- **Beef:**

☐ **0** ☐ **<1** ☐ **1 to 2** ☐ **3 to 4** ☐ **4** ☐ **> 1 time per week**

- **Animal fat:**

☐ **Never** ☐ **small part** ☐ **Medium part** ☐ **Great part**

- **Dairy product:**

☐ **Small part (a)** ☐ **Medium part (b)** ☐ **Great part (c)**

(a) Only one cup of milk or some other product (cheese, yogurt..) per day
(b) One cup of milk with some other product (cheese, yogurt..) per day
(c) More than 2 cups of milk per day or/with a big quantity of other product (cheese, yogurt..).

- **Total fruit, time/week:**

☐ **< 2** ☐ **2 to 6** ☐ **7 to 14**

- **Total vegetables, time/week:**

☐ **< 7** ☐ **7 to 13** ☐ **≥ 14**

- **Green tea consumption, cups/day:**

☐ **< 1** ☐ **1 to 3** ☐ **> 3**

5. Alcohol

☐ **Never** ☐ **Current user (for how many years and how many drink/day)**

☐ **Former user (drank and stopped for how many years and how many drink/day)**

6. Smoking

☐ **Non-smokers** ☐ **Current smokers (number of cigarettes/day and for how many years)**

☐ **Former smokers (number of cigarettes/day and for how many years)**
